# 硫利达嗪对肺癌PC9细胞的杀伤效应及其机制

**DOI:** 10.3779/j.issn.1009-3419.2015.12.03

**Published:** 2015-12-20

**Authors:** 理 龚, 毅 汪, 斯浩 童, 柳 刘, 伶 牛, 媛 袁, 扬漪 鲍

**Affiliations:** 1 230061 合肥，安徽医科大学第三附属医院（合肥市第一人民医院）肿瘤科 Department of Oncology, the Third Affiliated Hospital of Anhui Medical University, Hefei 230061, China; 2 230061 合肥，合肥市滨湖医院中心实验室 Central Laboratory of Hefei Binhu Hospital, Hefei 230061, China

**Keywords:** 肺肿瘤, 硫利达嗪, 凋亡, Caspase凋亡途径, Lung cancer, Thioridazine, Apoptosis, Caspase apoptotic pathway

## Abstract

**背景与目的:**

硫利达嗪作为一种吩噻嗪类抗精神疾病药物，近期研究显示其在体外可抑制多种肿瘤细胞的增殖，但对肺癌的作用尚未见报道。本实验以PC9细胞株为研究对象，旨在观察硫利达嗪对其杀伤效应以及探讨其可能的作用机制。

**方法:**

不同浓度的硫利达嗪作用PC9细胞后，四甲基偶氮唑蓝（methyl thiazolyltetrazolium, MTT）法检测细胞增殖率，流式细胞术检测细胞周期及细胞凋亡率，Western blot检测周期相关蛋白CyclinD1及凋亡相关蛋白Caspase-3、Caspase-8、Caspase-9、Bcl-2、Bax、Bcl-xl表达水平。

**结果:**

硫利达嗪可显著抑制PC9细胞的增殖，其抑制作用呈时间和浓度依赖性。流式结果显示：随着硫利达嗪药物浓度的增高，细胞发生不同程度的G_0_/G_1_期阻滞，细胞凋亡率明显增高。各实验组与对照组比较，差异有统计学意义（*P* < 0.05）。Western blot结果显示：与对照组比较，实验组CyclinD1、Bcl-2、Bcl-xl表达水平明显下调（*P* < 0.01），Bax表达水平明显上调（*P* < 0.01），Caspase-3、Caspase-8、Caspase-9活性显著增加（*P* < 0.01）。

**结论:**

硫利达嗪可显著抑制PC9细胞的增殖，其机制可能与其激活Caspase内外源性凋亡途径，下调CyclinD1、Bcl-2、Bcl-xl，上调Bax有关。

## Abstract (en)

**Background and objective:**

Recent research shows thioridazine which is a kind of phenothiazine antipsychotic drugs can inhibit the proliferation of various tumor cells *in vitro*, but the role of thioridazine on lung cancer has not been reported. So we choose PC9 cell lines as the research object, the aim is to oberve the killing effect of thioridazine on PC9 cells and investigate its possible mechanism.

**Methods:**

After being treated with different concentrations of thioridazine, the proliferation of PC9 cells was determined by methyl thiazolyltetrazolium (MTT) assay. Flow cytometry was used to measure the cell cycle distribution and apoptosis. The expressions of cell cycle-associated protein CyclinD1 and apoptosis-related proteins Caspase-3, Caspase-8, Caspase-9, Bcl-2, Bax and Bcl-xl were detected by Western blot.

**Results:**

The proliferation of PC9 cells was significantly inhibited by thioridazine in a dose-and time-dependent manner. Flow cytometry showed that cell cycle was arrested in G_0_/G_1_ phase and the apoptotic rates were significantly increased with the increasing concentration of thioridazine. Compared with the control group, the differences were statistically significant (*P* < 0.05). Western blot analysis showed that, compared with the control group, thioridazine reduced the expressions of CyclinD1, Bcl-2 and Bcl-xl (*P* < 0.01) and increased the expression of Bax (*P* < 0.01). In the mean time, thioridazine promoted the activities of Caspase-3, Caspase-8 and Caspase-9 (*P* < 0.01).

**Conclusion:**

The mechanism of thioridazine inhibiting the proliferation of PC9 cells may be related to stimulation of Caspase apoptotic pathway, down-regulation of CyclinD1, Bcl-2, Bcl-xl and up-regulation of Bax.

肺癌已成为我国发病率最高的恶性肿瘤，其中非小细胞肺癌（non-small cell lung cancer, NSCLC）约占80%-85%^[1]^，严重威胁着人们的生命健康。近年来针对NSCLC的治疗，主要包括化疗和靶向治疗，虽明显改善了进展期患者的生存，使其中位无进展生存期达8个月-12个月，但仍不能治愈疾病^[2]^。因此寻找新的药物和治疗手段非常必要。

硫利达嗪作为一种多巴胺受体阻断剂，可靶向作用于大脑中的多巴胺受体，缓解精神障碍所致的紧张与焦虑，曾被广泛应用于精神分裂症等精神疾病的治疗。近期研究却显示硫利达嗪在体外可抑制多种肿瘤细胞的增殖，如宫颈癌、乳腺癌、胃癌及肝癌等^[3-6]^，提示其潜在的抗肿瘤作用，但对NSCLC的作用尚未见报道。由于我国晚期肺腺癌患者中，表皮生长因子受体（epidermal growth factor receptor, *EGFR*）突变阳性率高达50%左右^[7]^，临床上针对这部分*EGFR*突变患者的靶向药物治疗如吉非替尼等也存在继发耐药的问题^[8]^，因此本课题以*EGFR*突变型NSCLC PC9细胞为研究对象，观察硫利达嗪对其增殖、凋亡以及细胞周期分布的影响，在此基础上研究其可能的作用机制，为治疗*EGFR*突变型NSCLC的新药研发提供实验依据。

## 1 材料与方法

### 1.1 材料

#### 1.1.1 细胞株与实验药物

PC9细胞株购自中国科学院上海细胞库，硫利达嗪购自美国Sigma公司。

#### 1.1.2 主要试剂与仪器

高糖（4, 500 mg/L葡萄糖）DMEM培养基购自美国Hyclone公司; 胎牛血清、二甲基亚砜（DMSO）、EDTA-胰酶购自美国Gibco公司; MTT粉购自Sigma公司; Annexin V-FICT/PI双染凋亡检测试剂盒以及细胞周期检测试剂盒购于贝博公司; 小鼠抗人CyclinD1、Bcl-2、Bcl-xl、Bax单克隆抗体购于Abcam公司; 兔抗人Caspase-3、Caspase-8和Caspase-9单克隆抗体购自Cell Signaling Technology公司; 二抗购于北京中杉金桥生物公司; 8000DH型二氧化碳培养箱购自美国Thermo公司; 680全自动酶标仪购自美国BIO-RAD公司; FACS Calibur流式细胞仪购自美国BD公司。

### 1.2 细胞培养与药物配制

PC9细胞用含10%胎牛血清、1%双抗（100 U/mL青霉素和100 μg/mL链霉素）的高糖DMEM培养基置于37 ℃、5%CO_2_的饱和湿度恒温培养箱中培养。1 d-2 d换液，用0.25%的胰酶消化传代，倒置显微镜下观察细胞生长情况，取对数生长期细胞进行实验。硫利达嗪原药用生理盐水、DMSO辅溶，配制成0.1 mol/L的储存液于-80 ℃冰箱分装保存。实验时稀释成工作液，DMSO终浓度小于0.1%。

### 1.3 MTT法检测细胞增殖抑制效应

取对数生长期PC9细胞，通过细胞计数调整细胞数为5×10^4^个/mL，100 μL每孔接种于96孔板中，实验组和对照组均设5个复孔，边缘孔以无菌PBS填充。37 ℃孵育24 h至细胞贴壁生长，实验组加入10 μmol/L、20 μmol/L、30 μmol/L、40 μmol/L、50 μmol/L的硫利达嗪，对照组加入培养基，分别于培养12 h、24 h、48 h后，每孔加入10 μL MTT（5 g/L），继续培养4 h，弃去上清，每孔加入100 μL DMSO，室温振荡10 min，酶标仪上检测各孔在490 nm处吸光度值（optical density, OD值）。按公式计算细胞存活率=（实验组OD均值/对照组OD均值）×100%，并用SPSS 16.0统计软件计算硫利达嗪的半数抑制浓度（half maximal inhibitory concentration, IC_50_）。

### 1.4 流式细胞术检测细胞周期变化

取对数生长期PC9细胞以1×10^5^个/孔接种于6孔板中，每孔2 mL体系，待细胞贴壁后换液。实验组分别加入浓度为15 μmol/L、20 μmol/L、25 μmol/L的硫利达嗪，对照组加培养基，实验与对照组均设3个复孔，继续培养24 h后，胰酶消化细胞，预冷PBS洗涤两遍，弃去上清，以预冷的70%乙醇4 ℃固定12 h。1, 600 r/min离心5 min，弃去固定液乙醇，PBS洗涤2遍，300 μLPBS重悬细胞后加入RNA酶20 μL于37 ℃水浴30 min，离心弃上清，加入400 μL碘化丙啶（propidium iodide, PI）染液，4 ℃避光孵育40 min，上流式细胞仪检测，实验重复3次。

### 1.5 流式细胞术检测细胞凋亡

取对数生长期PC9细胞以1×10^5^个/孔接种于6孔板中，每孔2 mL体系，待细胞贴壁后换液。实验组分别加入浓度为20 μmol/L、30 μmol/L、40 μmol/L的硫利达嗪，对照组加培养基，实验与对照组均设3个复孔，继续培养24 h后，消化收集细胞（包括上清中的细胞），PBS洗涤2遍，弃去上清，加入10 μL Annexin V和5 μL PI做标记，震荡混匀后室温避光孵育15 min，PBS洗2遍后加400 μL鞘液重悬，上流式细胞仪检测细胞凋亡情况。

### 1.6 Western blot检测周期及凋亡相关蛋白的表达

取对数生长期细胞，以对照组、硫利达嗪20 μmol/L、25 μmol/L、30 μmol/L处理组分别收集细胞。以蛋白裂解液提取总蛋白，BCA法蛋白定量后，灌胶，蛋白样品处理后上样，经12%SDS-PAGE凝胶电泳，电转印至PVDF膜上（220 mA、120 min），于5%脱脂奶粉中室温封闭1 h。加入一抗（1:1, 000稀释）孵育，4 ℃过夜，TBST冲洗3次，每次10 min，二抗（1:5, 000稀释）室温孵育1 h，TBST室温洗3次，每次10 min，ECL法发光、显影、定影。

### 1.7 统计学方法

所有试验均重复3次，采用SPSS 16.0统计软件进行数据分析。实验数据用Mean±SD表示，多组间数据比较采用单因素方差分析，两组之间比较采用*Dunnett-t*检验，采用Probit分析计算药物的IC_50_值。以*P* < 0.05为差异有统计学意义。

## 2 结果

### 2.1 硫利达嗪对NSCLC PC9细胞增殖的影响

MTT检测结果显示，硫利达嗪作用12 h、24 h、48 h后对PC9细胞的增殖均有抑制作用，其IC_50_分别为（45.851±2.59）μmol/L、（37.726±1.96）μmol/L和（32.594±1.73）μmol/L。各时间段内实验组与对照组比较，差异有统计学意义（*F*=362.7、439.6、911.1, *P* < 0.05）（图 1）。倒置光学显微镜（×200倍）下观察细胞形态，可见随着药物浓度的增加，正常贴壁细胞逐渐减少、细胞轮廓不清、体积缩小、间隙增加，细胞变圆变亮，漂浮状细胞增多（图 2）。

**1:**
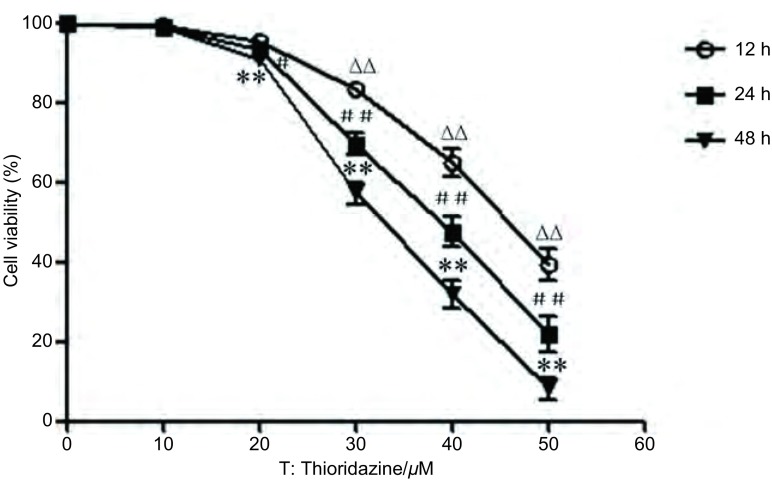
MTT法检测硫利达嗪对PC9细胞的增殖抑制效应。不同浓度硫利达嗪作用PC9细胞12 h、24 h、48 h后，分别用MTT法检测其细胞存活率。单因素方差分析，不同浓度硫利达嗪作用12 h后与对照组比较，^∆∆^*P* < 0.01；作用24 h后与对照组比较，^#^*P* < 0.05，^##^*P* < 0.01；作用48 h后与对照组比较，^**^P < 0.01。 Proliferation inhibitory effect of thioridazine on PC9 cells detected by MTT. The viability of PC9 cells treated with different concentrations of thioridazine for 12 h, 24 h and 48 h was detected by MTT assay. The statistical analysis was performed with one-way analysis of variance. ^∆∆^*P* < 0.01 compared with the control group after being treated with thioridazine for 12 h, ^#^*P* < 0.05, ^##^*P* < 0.01 compared with the control group after being treated with thioridazine for 24 h, ^**^P < 0.01 compared with the control group after being treated with thioridazine for 48 h.

**2:**
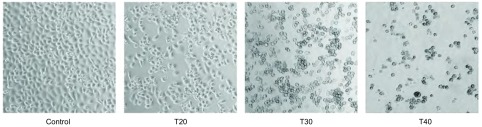
硫利达嗪作用后对PC9细胞形态学的影响（SP, ×200）。浓度为20 μmol/L、30 μmol/L、40 μmol/L的硫利达嗪作用于PC9细胞24 h后，观察其细胞形态。 Effect of cell morphology on PC9 cells by thioridazine (SP, ×200). The cell morphology was observed after being treated with different concentrations of thioridazine (20 μmol/L, 30 μmol/L, 40 μmol/L) for 24 h. T: Thioridazine/μM.

### 2.2 流式细胞术检测硫利达嗪对PC9细胞周期的影响

15 μmol/L、20 μmol/L、25 μmol/L的硫利达嗪作用于PC9细胞24 h后，G_0_/G_1_期细胞比例分别为（48.9±3.1）%、（59.7±2.8）%、（74.2±3.2）%，处于G_0_/G_1_期的细胞比例升高明显，即硫利达嗪可诱导PC9细胞发生G_0_/G_1_期阻滞，与对照组（40.0±2.5）%相比，差异有统计学意义（*F*=76.51, *P* < 0.05）（图 3）。

**3:**
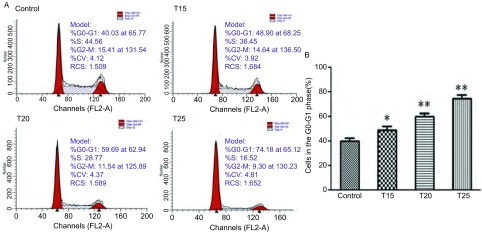
硫利达嗪作用24 h后对PC9细胞周期分布的影响。A：浓度为15 μmol/L、20 μmol/L、25 μmol/L的硫利达嗪作用于PC9细胞24 h后，流式细胞术检测其细胞周期分布情况；B：各实验组与对照组比较，差异有统计学意义。^*^P < 0.05，^**^P < 0.01。 Effect of thioridazine on the cell cycle distribution of PC9 cells for 24 h. A: Flow cytometry was used to measure the cell cycle distribution after treating with different concentrations of thioridazine for 24 h. B: The differences were statistically significant by compared with the control group.^*^P < 0.05, ^**^P < 0.01. T: Thioridazine/μM.

### 2.3 流式细胞术检测硫利达嗪对PC9细胞凋亡的影响

20 μmol/L、30 μmol/L、40 μmol/L的硫利达嗪作用于PC9细胞24 h后，促进细胞发生凋亡，其凋亡率分别为（9.3±2.1）%，（28.7±4.7）%，（50.0±5.4）%，与对照组相比，差异有统计学意义（*F*=104.7, *P* < 0.05）（图 4）。

**4:**
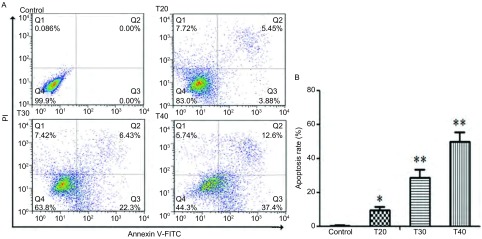
硫利达嗪作用24 h后对PC9细胞凋亡的影响。A：浓度为20 μmol/L、30 μmol/L、40 μmol/L的硫利达嗪作用24 h后，流式细胞术用Annexin V-FITC和PI双标检测细胞凋亡率；B：与对照组相比，各实验组细胞凋亡率明显上升。^*^P < 0.05，^**^P < 0.01。 Effect of thioridazine on the induction of apoptosis in PC9 cells for 24 h. A: After being incubated with different concentrations of thioridazine for 24 h, the apoptosis rate was analyzed using Annexin V-FITC and PI. B: Compared with the control group, each experiment group induced an increase in cell apoptosis rate. ^*^P < 0.05, ^**^P < 0.01. T: Thioridazine/μM.

### 2.4 Western blot检测周期及凋亡相关蛋白表达

Western blot结果显示：与对照组相比，随着硫利达嗪药物浓度的增加，周期蛋白CyclinD1表达下调（*F*=23.58, *P* < 0.01)，抗凋亡蛋白Bcl-2表达下调（*F*=16.87, *P* < 0.01)，Bcl-xl表达下调（*F*=51.47, *P* < 0.01)，促凋亡蛋白Bax表达上调（*F*=26.44, *P* < 0.01)。实验组Caspase-3、Caspase-8、Caspase-9活性较对照组增加（图 5）。

**5:**
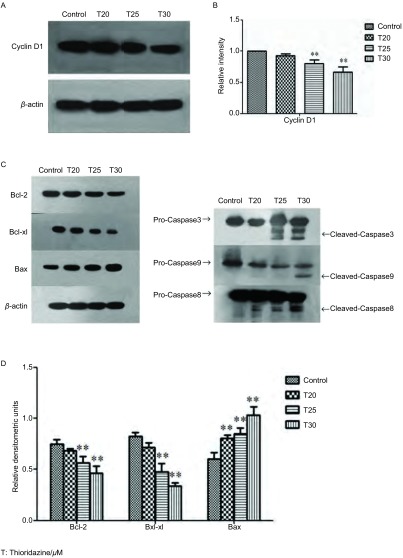
Western blot检测周期及凋亡相关蛋白水平的改变。A：20 μmol/L、25 μmol/L、30 μmol/L的硫利达嗪作用24 h后，Western blot检测周期相关蛋白CyclinD1的表达水平；B：与对照组相比，CyclinD1表达下调。^**^P < 0.01；C：20 μmol/L、25 μmol/L、30 μmol/L的硫利达嗪作用24 h后，Western blot检测凋亡相关蛋白Bcl-2、Bax、Bcl-xl、Caspase-3、Caspase-8、Caspase-9的表达水平；D：与对照组相比，抗凋亡蛋白Bcl-2、Bcl-xl表达下调，促凋亡蛋白Bax表达上调。^**^P < 0.01。 Expressions of cycle-and apoptosis-related proteins detected by Western blot. A: The expression of cell cycle-related protein CyclinD1 was detected by western blot after incubation with different concentrations of thioridazine for 24 h; B: Compared with the control group, thioridazine down-regulated the expression of CyclinD1. ^**^P < 0.01; C: The expressions of apoptosis-related proteins Bcl-2, Bax, Bcl-xl, Caspase-3, Caspase-8 and Caspase-9 were detected by western blot after incubation with different concentrations of thioridazine for 24 h; D: Compared with the control group, thioridazine down-regulated the expressions of Bcl-2, Bcl-xl and up-regulated the expression of Bax. ^**^P < 0.01. T: Thioridazine/μM.

## 3 讨论

*EGFR*突变是NSCLC中常见的基因突变类型，多项研究证实，*EGFR*基因突变与小分子酪氨酸激酶抑制剂（如吉非替尼、厄洛替尼等）的疗效关系密切。Tokumo等^[9]^对21例NSCLC患者的研究显示，发生*EGFR*突变的NSCLC患者对吉非替尼的治疗应答率显著高于EGFR未突变组。但酪氨酸激酶抑制剂在治疗过程中常易发生获得性耐药，发生耐药的患者后续治疗结果较差——故寻找新的药物来治疗*EGFR*突变型NSCLC十分必要。所以本实验选取*EGFR*突变型NSCLC细胞株PC9作为研究对象。

硫利达嗪作为一种吩噻嗪类抗精神疾病药物，主要用于精神分裂症、躁狂症等精神类疾病的治疗。随着研究的深入，多项研究表明硫利达嗪在体外有着显著的抗肿瘤作用。Mao等^[3]^发现硫利达嗪可通过阻断多巴胺受体介导的信号转导，从而显著诱导宫颈癌细胞SiHa发生凋亡，提示硫利达嗪可能作为一种新的抗肿瘤药物。Lu等^[6]^以肝癌细胞SNU449、LM3和Huh7为研究对象，发现硫利达嗪对肝癌细胞增殖的抑制作用呈剂量依赖性，可能与硫利达嗪诱导肝癌细胞发生G_0_/G_1_期阻滞以及抑制其干性基因CD133、OCT4和EpCam的表达有关。本实验结果也显示，硫利达嗪可显著抑制PC9细胞增殖，其抑制作用呈时间和浓度依赖性——随着药物的干预，PC9细胞皱缩变圆，活细胞数量显著降低。流式细胞术结果显示，25 μmol/L的硫利达嗪作用PC9细胞后，处于G_0_/G_1_期细胞比例最高，可达74.2%，较对照组40.0%明显增高，显示硫利达嗪可诱导PC9细胞发生G_0_/G_1_期阻滞。研究显示CyclinD1是调控细胞G_1_期的关键蛋白，它可以促进细胞周期由G_1_期进入S期，对细胞周期调控至关重要^[10]^。本实验Western blot显示硫利达嗪可使CyclinD1表达下调，从而抑制细胞进入S期的进程，导致大量细胞阻滞在G_0_/G_1_期。同时流式细胞术结果显示，20 μmol/L的硫利达嗪可诱导PC9细胞发生凋亡，凋亡率为9.33%，且随着药物浓度的增加，细胞凋亡率也明显上升，证实了硫利达嗪体外具有抗肿瘤作用。

凋亡是细胞内的程序性死亡，与肿瘤的发生、发展密切相关，Bcl-2家族蛋白与Caspase家族蛋白在细胞凋亡发生过程中发挥着重要作用^[11, 12]^。Bcl-2家族蛋白主要作用于细胞线粒体膜上，通过抗凋亡蛋白和促凋亡蛋白发挥着凋亡调控作用^[13]^。Bcl-2和Bcl-xl作为重要的抗凋亡因子，可通过BH3结构域与促凋亡蛋白形成异源二聚体，从而使其失活，保护细胞不进入凋亡程序; 同时Bcl-2和Bcl-xl也能通过抑制细胞色素C（Cytochrome C, Cyt C）的释放发挥抗凋亡作用^[14]^。促凋亡蛋白Bax则通过破坏线粒体膜的稳定性，促进线粒体释放Cyt C，从而诱发细胞凋亡^[15]^。Caspase家族在介导线粒体凋亡过程中发挥着重要作用，其中Caspase-8、Caspase-9是凋亡启动因子，Caspase-3是凋亡执行因子。线粒体释放到胞浆的Cyt C可通过与Apaf-1因子聚合从而激活Caspase-9内源性凋亡途径，继而激活下游的Caspase-3，导致Caspase级联反应诱发凋亡^[16]^。外源性凋亡途径中Caspase-8活化后几乎能激活所有级联反应下游的Caspase导致细胞凋亡^[17]^。

Mu等^[5]^证实硫利达嗪可通过激活Caspase途径诱导胃癌细胞凋亡发生; 雍敏等^[18]^研究发现硫利达嗪诱导卵巢癌细胞SKOV3、A2780凋亡，可能与其上调P53、Bax、胞质Cyt C、active Caspase-3表达，下调Bcl-2、线粒体Cyt C表达有关。这两项研究表明硫利达嗪诱导肿瘤细胞凋亡的机制可能与Bcl-2家族蛋白、Caspase家族蛋白有关。本实验Western blot结果提示，随着药物浓度的增加，PC9胞内促凋亡蛋白Bax表达上调，同时抗凋亡蛋白Bcl-2、Bcl-xl表达下调。与对照组相比，实验组胞内Caspase-3、Caspase-8和Caspase-9活性均明显增加。以上结果与Mu、雍敏等研究结果相似，表明硫利达嗪诱导PC9细胞凋亡，可能是通过激活Caspase内外源性凋亡途径，下调抗凋亡蛋白Bcl-2、Bcl-xl，上调Bax来实现的。

综上所述，硫利达嗪可显著诱导*EGFR*突变型NSCLC细胞株PC9发生细胞周期阻滞和凋亡，其抗肿瘤生物学活性提示其潜在的临床价值和应用前景。但目前硫利达嗪抗肿瘤细胞的机制仍未完全明确，其抑瘤效应是否与多巴胺受体表达有关，以及其联合放化疗或靶向药物治疗是否具有协同作用仍需进一步深入研究。
